# Clinical aspects and cytokine response in severe H1N1 influenza A virus infection

**DOI:** 10.1186/cc9324

**Published:** 2010-11-09

**Authors:** Natalia Hagau, Adriana Slavcovici, Daniel N Gonganau, Simona Oltean, Dan S Dirzu, Erika S Brezoszki, Mihaela Maxim, Constantin Ciuce, Monica Mlesnite, Rodica L Gavrus, Carmen Laslo, Radu Hagau, Magda Petrescu, Daniela M Studnicska

**Affiliations:** 1 University Emergency County Hospital of Cluj, Clinicilor 3-5, 400006 Cluj-Napoca, Romania; 2 Teaching Hospital of Infectious Disease Cluj-Napoca, Iuliu Moldovan 23, 400348 Cluj-Napoca, Romania; 3 Memorial Hospital of Rhode Island, Brown University, 111 Brewster Street, Pawtucket, RI 02860, USA

## Abstract

**Introduction:**

The immune responses in patients with novel A(H1N1) virus infection (nvA(H1N1)) are incompletely characterized. We investigated the profile of Th1 and Th17 mediators and interferon-inducible protein-10 (IP-10) in groups with severe and mild nvA(H1N1) disease and correlated them with clinical aspects.

**Methods:**

Thirty-two patients hospitalized with confirmed nvA(H1N1) infection were enrolled in the study: 21 patients with nvA(H1N1)-acute respiratory distress syndrome (ARDS) and 11 patients with mild disease. One group of 20 patients with bacterial sepsis-ARDS and another group of 15 healthy volunteers were added to compare their cytokine levels with pandemic influenza groups. In the nvA(H1N1)-ARDS group, the serum cytokine samples were obtained on admission and 3 days later. The clinical aspects were recorded prospectively.

**Results:**

In the nvA(H1N1)-ARDS group, obesity and lymphocytopenia were more common and IP-10, interleukin (IL)-12, IL-15, tumor necrosis factor (TNF)α, IL-6, IL-8 and IL-9 were significantly increased versus control. When comparing mild with severe nvA(H1N1) groups, IL-6, IL-8, IL-15 and TNFα were significantly higher in the severe group. In nonsurvivors versus survivors, IL-6 and IL-15 were increased on admission and remained higher 3 days later. A positive correlation of IL-6, IL-8 and IL-15 levels with C-reactive protein and with > 5-day interval between symptom onset and admission, and a negative correlation with the PaO_2_:FiO_2 _ratio, were found in nvA(H1N1) groups. In obese patients with influenza disease, a significant increased level of IL-8 was found. When comparing viral ARDS with bacterial ARDS, the level of IL-8, IL-17 and TNFα was significantly higher in bacterial ARDS and IL-12 was increased only in viral ARDS.

**Conclusions:**

In our critically ill patients with novel influenza A(H1N1) virus infection, the hallmarks of the severity of disease were IL-6, IL-15, IL-8 and TNFα. These cytokines, except TNFα, had a positive correlation with the admission delay and C-reactive protein, and a negative correlation with the PaO_2_:FiO_2 _ratio. Obese patients with nvA(H1N1) disease have a significant level of IL-8. There are significant differences in the level of cytokines when comparing viral ARDS with bacterial ARDS.

## Introduction

Originating from Mexico and spreading initially in the United States and Canada, a novel influenza A(H1N1) virus infection (nvA(H1N1)) of swine origin spread globally during spring 2009 to mid-February 2010. Rates of hospitalization and death have varied widely according to country [1].Among hospitalized patients 9 to 31% have been admitted to intensive care units (ICUs) where the rate of death was 14 to 46%[2-6].

In Romania the pandemic wave lasted from September 2009 to February 2010, reaching a peak in December. The Romanian Ministry of Health reported 7,008 confirmed cases of nvA(H1N1) influenza, the death rate being 1.9%.

Primary influenza pneumonia had a high mortality rate during pandemics not only in immune-compromised individuals and patients with underlying co-morbid conditions, but also in young healthy adults [7].

During nvA(H1N1) virus infection, experimental and clinical studies have identified dysregulated systemic inflammation as an important pathogenetic mechanism correlating with severity and progression of the disease [8,9]. The role of most immune responses in controlling and clearance of H1N1 influenza A or its contribution to severe respiratory compromise is not well known.

To and colleagues found higher plasma levels of proinflammatory cytokines and chemokine in the group of patients with acute respiratory distress syndrome (ARDS) caused by viral A(H1N1) influenza, throughout the initial 10 days after symptom onset [8]. Bermejo-Martin and colleagues found that mediators involved in the development of Th17 cells (IL-6, IL-8, IL-9, IL-17), Th1 cells (TNFα, IL-15, IL-12p70) and type II interferon (IFNγ) had high systemic levels in hospitalized patients with nvA(H1N1) influenza [9]. The detrimental or beneficial role of these cytokines in severe illness is not known.

The aim of our study was to further investigate the profile of Th1 and Th17 mediators and interferon-inductible protein-10 (IP-10), an innate-immunity mediator, as early host response in a group of critical and noncritical hospitalized patients with nvA(H1N1) from Cluj-Napoca, Romania, and to correlate them with the clinical aspects.

## Materials and methods

### Patients and controls

The study was performed between October 2009 and February 2010 in the ICUs of the Emergency County Clinical Hospital and of the Teaching Hospital of Infectious Diseases, Cluj-Napoca, Romania.

Thirty-two patients hospitalized with nvA(H1N1) infection were enrolled in the study: 21 patients with nvA(H1N1)-ARDS, and 11 patients with nvA(H1N1)-mild disease. Additionally, 20 patients with bacterial sepsis-ARDS were included and served to compare the cytokine levels between the nvA(H1N1)-ARDS group and the bacterial sepsis-ARDS group.

The study protocol was approved by the Ethics Committee for Clinical Research of the University of Medicine and Pharmacy 'Iuliu Hatieganu' Cluj Napoca and the hospital authority. Informed consent was obtained from each patient or their legal representative.

The inclusion criteria were age > 16 years, symptoms compatible with influenza and confirmed nvA(H1N1) virus, bacterial severe sepsis with ARDS, and informed consent. The exclusion criteria were age < 16 years, known infection by human immunodeficiency virus, patients with other respiratory viral infections, bacterial sepsis without ARDS-syndrome, and refusal to consent.

The control group included 15 healthy volunteers without chronic or acute disease.

Data were recorded prospectively by investigators at each hospital. The following data were recorded: age, sex, pregnancy, underlying diseases (chronic obstructive pulmonary disease, asthma, diabetes, chronic heart failure, chronic renal failure, cirrhosis, immunosuppression), obesity defined as body mass index > 30, and the time in days from symptom onset to hospital admission. Hematological, biochemical and microbiological results were included in the database. The extension of lung infiltrates on chest X-ray scan was registered as the number of quadrants involved. The severity and prognosis of the illness was assessed in adults using the Acute Physiology and Chronic Health Evaluation II (APACHE II) score and the Sepsis-related Organ Failure Assessment (SOFA) score. ARDS was defined using the 1994 American-European Consensus Conference definitions [10]. The pulmonary dysfunction score was based on the PaO_2_:FiO_2 _ratio, ranging from 0 to 3 where grade 0 represented a ratio less or equal to 250; grade 1, a ratio ranging from 250 to 175; grade 2, a ratio ranging from 100 to 175; and grade 3, a ratio less or equal to 100[11].

A(H1N1) influenza virus presence was confirmed by testing nasopharyngeal swabs or bronchoalveolar lavage specimens with real-time PCR (commercial kits: Full Velocity SYBR Green QRT-PCR/SuperScript III Platinum One-Step Quantitative RT-PCR Taqman; Invitrogen Corporation, Carlsbad, California, USA) at The National Influenza Centre of Cantacuzino Institute, Bucharest, Romania.

### Cytokine and chemokine quantification

In patients with nvA(H1N1)-mild disease, the serum samples were taken on hospital admission. In patients with nvA(H1N1)-ARDS infection, the serum samples were taken on admission to the ICU and 3 days later to determine cytokine kinetics. The installation of ARDS, either viral or bacterial, in the course of the disease determined the time of admission to the ICU. In patients with bacterial sepsis-related ARDS, the serum samples were taken on admission to the ICU.

The enrolled patients and the healthy volunteers gave whole blood, which was clotted for 30 minutes at 37°C and stored at -70°C until use. The resulting serum was used for cytokine determination.

Seven different serum cytokines (IL-6, IL-8, IL-12p70, IL-15, IL-17, TNFα and IFN-γ) were measured with Luminex 200 (Luminex Corporation, Austin, TX, USA) using a multiplex cytokine kit along with the assay performed in accordance with the manufacturer's instructions (R&D Systems, Minneapolis, MN, USA). Additionally, we used ELISA kits for quantitative determination of the two cytokines IL-9 and IP-10 (Quantikine; R&D Systems).

### Statistical analysis

Subjects were stratified into three groups: 11 patients with nvA(H1N1)-mild disease, 21 patients with nvA(H1N1)-ARDS, and 20 patients with bacterial sepsis-ARDS.

Descriptive statistics included means and standard deviations or medians and interquartile ranges for continuous variables of normal and non-normal distributions. Clinical and biochemical characteristics and cytokine levels were compared. The Fisher exact test and the chi-square test were used for categorical variables. The Mann-Whitney U test was used for nonparametric variables. The Wilcoxon test (nonparametric test) was used to compare two paired groups. The association between nonparametric variables was determined by the Spearman correlation coefficient (*r*). Any value of *P *< 0.05 was considered statistically significant. GraphPad Prism version 5.03 Software for Windows (GraphPad Software, La Jolla, California, USA) was used.

## Results

A total of 32 patients with confirmed nvA(H1N1) infection and 20 patients with bacterial sepsis-ARDS were enrolled over the study period. Their demographic, co-morbidities and clinical characteristics are presented in Table 1. Patients in the nvA(H1N1)-ARDS group were significantly older than those in the nvA(H1N1)-mild disease group (median age 42 years vs. 33 years, *P *= 0.009). Obesity was more common in the nvA(H1N1)-ARDS group. The median interval between onset of illness and admission was 6 days (interquartile range 3.5 to 8.5) in the nvA(H1N1)-ARDS group and 2 days (interquartile range 2 to 3) in the mild disease group (*P *< 0.001) (Table 1). All the patients with nvA(H1N1) virus infection presented symptoms of acute respiratory viral infection on admission. The median length of hospital stay was higher in the nvA(H1N1)-ARDS group compared with the mild disease group (11 days vs. 6 days, *P *< 0.001). All patients with nvA(H1N1) virus infection received oseltamivir on admission: the standard dose (150 mg/day) was administered for patients with mild disease, and a higher dose (300 mg/day) was used for nvA(H1N1)-ARDS patients. During the ICU hospitalization, critical patients with influenza virus infection (ARDS) received corticosteroid therapy (hydrocortisone or methylprednisolone). In agreement with our protocol, empirical antibiotics were started on admission.

**Table 1:** Demographic, co-morbidities and clinical characteristics of the patients

Characteristics of patients	All patients with nvA(H1N1) infection (*n *= 32)	nvA(H1N1)-ARDS group (*n *= 21)	nvA(H1N1)-mild disease group (*n *= 11)	*P *value^a^	ARDS bacterial sepsis group (*n *= 20)	*P *value^b^
Age (years)	37 (30.7 to 52)	42 (33.5 to 55.5)	33 (18 to 35)	0.009	57 (38.5 to 66)	0.12
Sex ratio (male/female)	16/16	9/12	7/4	0.4	8/12	1
Underlying disease						
Cardiovascular disease	8 (25%)	6/21	2/11	0.6	8/20	0.5
Asthma/COPD	10 (31.25%)	8/21	2/11	0.4	12/20	0.2
Obesity (BMI > 30)	13 (40.6%)	12/21	1/11	0.01	3/20	0.01
Type 1 or 2 diabetes	2 (6.3%)	2/21	0		1/20	1
Esquizophrenia	3 (9.3%)	3/21	0		0	
Cancer	3 (9.3%)	1/21	2/11	0.2	6/20	0.044
Pregnancy	3 (9.3%)	3/21	0		0	
Interval between symptom onset and hospital admission (days)	4.5 (2 to 7)	6 (3.5 to 8.5)	2 (2 to 3)	0.001	2 (0 to 3)	0.01
Presenting symptoms						
Fever > 38°C	28 (87.5%)	18/21	10/11	1	18/20	1
Cough	32 (100%)	21/21	11/11		2/20	
Dyspnea	25 (78%)	21/21	4/11		20/20	
Myalgia	24 (75%)	14/21	10/11	0.2	0	
SOFA score		6.1 (± 3.29)			7.2 (± 4.2)	0.5
APACHE II score	12.6 (± 6.62)	15.14 (± 6.36)			16.3 (± 6.03)	0.5
Respiratory condition						
SaO_2 _< 94%	23 (71.8%)	21/21	2/11		20/20	
PaO_2_:FiO_2 _ratio		1.96 (± 0.83)	NA		1.72 (± 0.68)	0.28
Mechanical ventilation	11 (34.3%)	11/21	0		12/20	0.7
Non-invasive ventilation	10 (31.3%)	10/21	0		8/20	0.7
Initial chest X-ray scan						
Extensive bilateral multilobar infiltrates	21 (65.6%)	21/21	0		20/20	
Complications						
Secondary bacterial pneumonia	3 (9.4%)	2/21	1/11		4/20	0.4
Pneumothorax	3 (9.4%)	3/21	0		2/20	1
Encephalitis	1 (3.1%)	1/21	0		0	
Acute renal failure requiring renal replacement therapy	4 (12.5%)	4/21	0		3/20	1
Length of hospital stay (days)	10 (7 to 12.7)	11 (10 to 14)	6 (6 to 9)	< 0.001	18 (13 to 41)	0.005
ICU length of stay (days)	4.5 (1 to 7.5)	7 (5 to 9)	0		16.5 (1 to 33)	0.3
In-hospital death	7 (21.8%)	7/21	0		3/20	0.2

Data presented as median (interquartile range), number (%) of patients, or mean (± standard deviation), unless otherwise indicated. APACHE, Acute Physiology and Chronic Health Evaluation; ARDS, acute respiratory distress syndrome; BMI, body mass index; COPD, chronic obstructive pulmonary disease, ICU, intensive care unit; NA, not applicable; nvA(H1N1), novel A(H1N1) virus; PaO_2_/FiO_2_, pressure of oxygen in arterial blood/fraction of inspired oxygen; SOFA, Sepsis-related Organ Failure Assessment.

^a ^differences in baseline characteristics between nvA(H1N1)-ARDS and nvA(H1N1)-mild disease (the Fisher exact test was used for categorical variables and Mann Whitney test for continuous variables)

^b ^differences in baseline characteristics between nvA(H1N1)-ARDS and bacterial sepsis-ARDS (the Fisher exact test was used for categorical variables and Mann Whitney test for continuous variables)

Among 21 patients with nvA(H1N1)-ARDS, four developed acute renal failure requiring renal replacement therapy, two developed secondary bacterial pneumonia and three developed pneumothorax (Table 1). Ten patients from the nvA(H1N1)-ARDS group received non-invasive ventilation and 11 patients received mechanical ventilation.

Pregnancy was another risk factor for nvA(H1N1)-ARDS infection and ICU admission (3/21 cases; Table 1). Two pregnant women were in the third trimester and one was in the second trimester. No underlying disease was noted. The range interval after symptom onset and ICU admission was 3 to 7 days. Caesarean delivery was necessary in two cases. All pregnant women required respiratory support (two invasive and one non-invasive) during hospitalization and all survived.

Seven patients died in the nvH1N1-ARDS group. Histopathological changes were similar in all cases: tracheitis, bronchitis with focal squamous metaplasia, necrotizing bronchiolitis, emphysema, extensive diffuse alveolar damage associated with alveolar hemorrhage and marked hyaline membrane formation, fibrosis and granulocyte pulmonary infiltrates. Pulmonary thromboemboli with focal infarcts were observed in three cases.

The lymphocyte count was significantly lower in the nvA(H1N1)-ARDS group than in the mild disease group (*P *= 0.011) (Table 2). Comparing laboratory abnormalities on hospital admission we found that patients with nvA(H1N1)-ARDS were more likely to have elevated levels of serum lactate dehydrogenase, alanine and aspartate aminotransferase (*P *< 0.001, *P *= 0.049 and *P *< 0.001, respectively) than patients with nvA(H1N1)-mild disease (Table 2).

**Table 2:** Laboratory characteristics of the patients

Laboratory characteristic	All patients with nvA(H1N1) infection (*n *= 32)	nvA(H1N1)-ARDS group (*n *= 21)	nvA(H1N1)-mild disease group (*n *= 11)	*P *value^a^	ARDS bacterial sepsis group (*n *= 20)	*P *value^b^
Leukocyte count (x10^3^/μl)	9.8 (7.5 to 11.8)	9.7 (8 to 12.3)	9.9 (6 to 10.5)	0.41	14.2 (8.2 to 20.5)	0.047
Lymphocyte count (x10^3^/μl)	1.05 (0.7 to 1.87)	0.9 (0.6 to 1.3)	1.6 (0.9 to 2.5)	0.011	1.38 (1.12 to 2.9)	0.009
Platelet count (x10^3^/μl)	218 (180 to 270)	218 (169 to 288)	218 (180 to 270)	0.7	241 (142.8 to 298)	0.8
C-reactive protein (mg/dl)	4.8 (1.2 to 9.6)	5.35 (4 to 10.2)	1.2 (0.6 to 4.4)	0.004	9.8 (5.75 to 22)	0.05
Procalcitonin (ng/ml)	0.23 (0.05 to 0.85)	0.39 (0.11 to 1.12)	0.06 (0.05 to 0.06)	0.003	6.3 (4.6 to 11.5)	< 0.001
LDH (IU/l)	698 (360 to 980)	890 (618 to 1134)	360 (250 to 400)	< 0.001	547 (408 to 812)	0.07
ALT (IU/l)	30.5 (17 to 80.75)	43 (21 to 92.5)	22 (15 to 30)	0.049	25 (13.5 to 50.75)	0.09
AST (IU/l)	42 (20 to 98)	95 (32.5 to 164)	17 (16 to 28)	< 0.001	25 (20 to 656)	0.003
Total protein level (mg/dl)	6.75 (6 to 7.55)	6.2 (5.8 to 7)	8 (7 to 8)	< 0.001	5.4 (4.8 to 6.4)	0.015
Creatinine level (mg/dl)	0.96 (0.6 to 1.17)	1.1 (0.9 to 1.25)	0.6 (0.5 to 0.8)	< 0.001	1.55 (1 to 2.37)	0.11

Data presented as median (interquartile range). ALT, alanine aminotransferase; ARDS, acute respiratory distress syndrome; AST, aspartate aminotransferase; LDH, lactate dehydrogenase; nvA(H1N1), novel A(H1N1) virus. ^a^Comparison between nvA(H1N1)-ARDS and nvA(H1N1)-mild disease (Mann-Whitney U test). ^b^Comparison between nvA(H1N1)-ARDS and bacterial sepsis-ARDS (Mann-Whitney U test).

Twenty patients with bacterial sepsis-ARDS were included to compare the cytokine levels in viral and bacterial ARDS. Immune suppression (six patients with cancer) was more common in the bacterial sepsis-ARDS group (*P *= 0.044). The mean (standard deviation) APACHE II score, SOFA score and PaO_2_:FiO_2 _ratio were similar in both groups (Table 1). The leukocyte count, C-reactive protein and procalcitonin levels were higher in the bacterial ARDS group than in the nvA(H1N1)-ARDS group (*P *= 0.047, *P *= 0.05 and *P *< 0.001, respectively) (Table 2).

**Figure 1:**
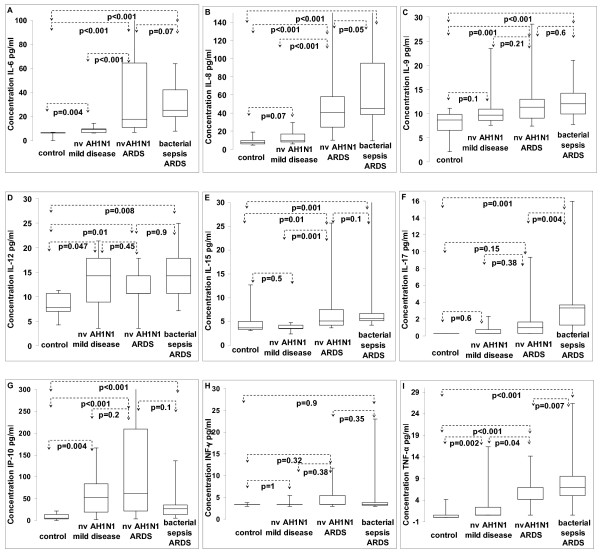
**Initial serum levels of cytokines in the four groups**. The Mann-Whitney U test was used to compare cytokine levels: **(A) **IL-6. **(B) **IL-8. **(C) **IL-9. **(D) **IL-12. **(E) **IL-15. **(F) **IL-17. **(G) **interferon-inductible protein-10 (IP-10). **(H) **IFNγ. **(I) **TNFα. Data presented as median, quartiles, and range. ARDS, acute respiratory distress syndrome; nvA(H1N1), novel A(H1N1) virus.

The results of the cytokine profile are shown in Figure 1. At admission, only IL-6, IL-12, IP-10 and TNFα were significantly higher in the mild disease group than in the control group. Except for IL-17 and IFNγ, all cytokine levels were higher in critical patients with nvA(H1N1)-ARDS than in the control group. Compared with the mild disease group, significantly higher levels of IL-6, IL-8, IL-15 and TNFα were found in the nvA(H1N1)-ARDS group (*P *< 0.001, *P *< 0.001, *P *< 0.001 and *P *< 0.05, respectively). Compared with controls, the levels of IL-6, IL-8, IL-9, IL-15, IL-17, IP-10 and TNFα were significantly elevated in the bacterial sepsis-ARDS group. Levels of IL-8, IL-17 and TNFα were significantly higher in the bacterial-ARDS group versus the nvA(H1N1)-ARDS group (*P *= 0.05, *P *= 0.004 and *P *= 0.011, respectively; Figure 1).

Patients with pandemic influenza virus (severe ARDS and mild disease) were stratified according to the interval between symptom onset and admission. Levels of IL-6, IL-8, IL-15 and IFNγ were significantly higher in patients with delayed admission, > 5 days after symptom onset (*P *= 0.006, *P *= 0.037, *P *= 0.013 and *P *= 0.027, respectively) (Table 3).

**Table 3:** Cytokine levels according to interval between symptom onset and admission in 32 hospitalized nvA(H1N1) patients

	Interval		
	
Cytokine (pg/ml)	1 to 5 days	6 to 14 days	*P *value^a^
IL-6	9.8 (7.1 to 14.65)	18 (13.76 to 84.47)	0.006
IL-8	16.63 (9.4 to 41.12)	39.89 (20.62 to 79.84)	0.034
IL-9	9.9 (9.1 to 13.1)	11.5 (8.55 to 12.85)	1
IL-12	10.67 (7.11 to 17.8)	10.67 (8.89 to 17.8)	0.9
IL-15	3.9 (3.6 to 5)	6 (4.15 to 11.15)	0.013
IL-17	0.56 (0.323 to 0.98)	0.323 (0.323 to 3.68)	0.6
IP-10	66.85 (21.6 to 166)	60 (15.21 to 163.7)	0.7
IFNγ	3.4 (2.965 to 3.4)	3.822 (3.4 to 5.625)	0.027
TNFα	4.15 (0.596 to 6.964)	4.15 (0.59 to 9.5)	0.3

Data presented as median (interquartile range). IP-10, interferon-inductible protein-10; nvA(H1N1), novel A(H1N1) virus. ^a^The Mann-Whitney test was used to compare the cytokine levels.

Serum cytokine levels over time (3 days after admission and antiviral treatment) showed a decrease of IL-6, IP-10, TNFα, IFNγ and IL-17 in critical patients with nvA(H1N1)-ARDS (Table 4). Serum cytokine levels over time in nvA(H1N1)-ARDS survivors showed a significant decrease of IL-6, IP-10 and TNFα (Table 5). In nonsurvivors versus survivors from the nvA(H1N1)-ARDS group, the levels of IL-6 and IL-15 on admission and 3 days after were significantly higher (Table 6). IL-17 was higher in nonsurvivors 3 days after admission (Table 6).

**Table 4:** Serum cytokine levels over time in the nvA(H1N1)-ARDS group (21 patients)

Cytokine(pg/ml)	P1^a^	P2^b^	*P *value^c^
IL-6	18 (10.41 to 64.88)	11 (8.09 to 23.77)	0.013
IL-8	40.14 (20.62 to 66.7)	24.47 (16.52 to 61.6)	0.18
IL-9	11.3 (9.05 to 14.25)	11.3 (9.65 to 14)	0.76
IL-12	10.67 (8.89 to 16.02)	14.24 (10.34 to 14.24)	0.9
IL-15	5.1 (4.15 to 8.35)	5 (3.9 to 7.7)	0.65
IL-17	0.98 (0.323 to 2.66)	0.323 (0.323 to 1.25)	0.049
IP-10	61.42 (20.5 to 274.5)	31.23 (9.19 to 49.19)	0.003
TNFα	4.15 (2.37 to 6.96)	0.596 (0.596 to 2.11)	0.001
IFNγ	3.4 (3.18 to 5.43)	3.4 (2.96 to 3.82)	0.041

Data presented as median (interquartile range). IP-10, interferon-inductible protein-10; nvA(H1N1), novel A(H1N1) virus. P1^a^Serum cytokine levels at admission.

P2^b^Serum cytokines levels 3 days after admission. ^c^The Wilcoxon test was used to compare the cytokine levels.

**Table 5:** Serum cytokine levels over time in nvA(H1N1)-ARDS survivors (14 patients)

Cytokine (pg/ml)	P1^a^	P2^b^	*P *value^c^
IL-6	14.76 (9.69 to 21.54)	8.73 (7.9 to 11.25)	0.02
IL-8	36.95 (16.75 to 41.49)	22.84 (14.6 to 46.86)	0.29
IL-9	10.6 (8.95 to 12.33)	11.3 (8.97 to 12.53)	0.45
IL-12	10.67 (7.11 to 14.24)	14.24 (9.78 to 15.13)	0.19
IL-15	4.55 (4.025 to 5.575)	4.7 (3.6 to 5.325)	0.4
IL-17	0.98 (0.323 to 1.146)	0.323 (0.323 to 0.323)	0.07
IP-10	57.26 (21.05 to 380.7)	29.67 (9.56 to 42.87)	0.01
TNFα	4.15 (3.26 to 6.96)	0.596 (0.596 to 1.485)	0.008
IFNγ	3.4 (2.965 to 3.92)	3.4 (2.965 to 3.925)	0.3

Data presented as median (interquartile range). IP-10, interferon-inductible protein-10; nvA(H1N1), novel A(H1N1) virus. P1^a^Serum cytokine levels at admission. P2^b^Serum cytokine levels 3 days after admission. ^c^The Wilcoxon test was used to compare cytokine levels over time..

**Table 6:** Serum cytokine levels at admission and 3 days later in nvA(H1N1)-ARDS group survivors versus nonsurvivors

Cytokine (pg/ml)	P1^a^			P2^b^		
	
	Survivors	Nonsurvivors	*P *value^c^	Survivors	Nonsurvivors	*P *value^c^
IL-6	14.76 (9.69 to 21.54)	64.76 (23.17 to 197.4)	0.025	8.73 (7.91 to 11.25)	26.9 (12 to 51.36)	0.005
IL-8	36.95 (16.75 to 41.49)	75.41 (36 to 148.3)	0.07	22.84 (14.6 to 46.86)	61 (17.44 to 89.8)	0.2
IL-9	10.6 (8.95 to 12.33)	13 (10.4 to 24.7)	0.12	11.3 (8.97 to 12.53)	14.5 (10.1 to 16.8)	0.2
IL-12	10.67 (7.11 to 14.24)	14.24 (10.67 to 17.8)	0.07	14.24 (9.78 to 15.13)	10.67 (10 to 14.24)	0.2
IL-15	4.55 (4.025 to 5.575)	10.6 (6.18 to 14.3)	0.006	4.7 (3.6 to 5.325)	10.4 (5.3 to 18.4)	0.012
IL-17	0.98 (0.323 to 1.146)	0.323 (0.323 to 4.373)	0.8	0.323 (0.323 to 0.323)	1.2 (0.323 to 1.645)	0.026
IP-10	57.26 (21.05 to 380.7)	99.87 (8.055 to 199.6)	0.9	29.67 (9.56 to 42.87)	40.2 (6.6 to 93.71)	0.5
TNFα	4.15 (3.262 to 6.964)	4.15 (0.596 to 11.89)	0.9	0.596 (0.596 to 1.485)	0.596 (0.596 to 2.12)	0.4
IFNγ	3.4 (2.965 to 3.925)	5.43 (3.4 to 5.819)	0.19	3.4 (2.965 to 3.925)	3.7 (3 to 3.82)	0.5

Data presented as median (interquartile range). IP-10, interferon-inductible protein-10; nvA(H1N1), novel A(H1N1) virus. P1^a^Serum cytokine levels at admission. P2^b^Serum cytokines levels 3 days after admission. ^c ^comparison between survivors and nonsurvivors (Mann-Whitney U test).

Correlation between cytokine levels and clinical or laboratory characteristics in patients with confirmed nvA(H1N1) infection was determined by Spearman correlation coefficient. We found significant correlation of IL-6, IL-8 and IL-15 levels with C-reactive protein (*r *= 0.67, *P *< 0.001; *r *= 0.5, *P *= 0.003; and *r *= 0.48, *P *= 0.005, respectively), with PaO_2_:FiO_2 _ratio (*r *= -0.556, *P *= 0.001; *r *= -0.574, *P *< 0.001; and *r *= -0.614, *P *< 0.001, respectively) and with interval between symptom onset and hospital admission (*r *= 0.51, *P *= 0.002; *r *= 0.41, *P *= 0.019; and *r *= 0.48, *P *= 0.004, respectively).

IL-8 was significantly higher (*P *= 0.013) in obese versus nonobese patients with nvA(H1N1) infection.

## Discussion

In this study we presented the cytokine profiles following nvA(H1N1) infection in 32 hospitalized patients (11 mild and 21 severe disease) and the cytokine profiles found in 20 cases of bacterial sepsis.

The patients with severe nvA(H1N1) disease were younger than the patients with bacterial sepsis (no statistical significance). Similarly to other study groups, we found that obesity was more common in the nvA(H1N1) ARDS group, suggesting it may be a risk factor for complications and admission to the ICU[2,5,6]. Laboratory findings in the same group of patients include lymphocytopenia and elevation in levels of alanine aminotransferase, aspartate aminotransferase, lactate dehydrogenase and creatinine - as in other patient groups with novel influenza virus infection [4,6]. In contrast, the bacterial-ARDS group presented no lymphocytopenia, lower elevation in serum liver enzymes and higher levels of C-reactive protein and procalcitonin. No significant differences were found between bacterial and viral ARDS groups in SOFA and APACHE II scores at admission. The pulmonary histopathological findings in nvA(H1N1)-ARDS nonsurvivors were similar to other fatal cases of nvA(H1N1) virus infection [12,13].

Installation of ARDS in the course of the disease was the moment of blood sampling for cytokine measurements. There was a difference regarding the time of symptom onset and hospital admission between the severe and mild groups of nvA(H1N1) disease that could affect the comparison of cytokine levels between the two groups. For this reason we not only compared the cytokine levels between mild and severe disease, but also mixed the patients with nvA(H1N1)-mild and severe disease and compared the level of cytokines according to the interval between symptom onset and admission (first interval 1 to 5 days, second interval 6 to 14 days). We found that not all cytokines had the same behavior against the time of symptom onset and admission.

The pattern of immune response in patients with nvA(H1N1) virus infection is incompletely characterized. CD4^+ ^T cells are known to play an important role in the initiation of immune responses by providing help to other cells. T-helper cells could be divided into subsets: Th1, Th2 and Th17.

Th1 cells mainly develop following infections by intracellular bacteria and some viruses [14]. The mediators involved in the development of Th1 are IL-12, IFNγ, IL-15, IL-18 and TNFα.

IL-12 bridges the early nonspecific innate immunity and the subsequent antigen-specific adaptative immunity [15]. IL-12 was shown to inhibit apoptosis of T cells [16] and of dendritic cells [17]. Alveolar macrophages have a functional IL-12 receptor, and virus-infected macrophages in the presence of IL-12 might be protected from apoptosis limiting viral clearance [18]. Apoptosis of virus-infected cells was shown to be an effective mechanism for viral clearance [19]. Bermejo-Martin and colleagues reported more significant IL-12 results in the critical A(H1N1) group of patients [9]. In our study, IL-12 is significantly higher in the nvA(H1N1)-mild disease group and in the nvA(H1N1)-ARDS group versus the control group and is not significantly higher in the bacterial ARDS group.

IL-15 plays a critical role in protecting CD8^+ ^T cells from apoptosis during the contraction phase following microbial infection [20,21]. The CD8^+ ^T cells surviving in the presence of IL-15 might be pathogenic in lung injury following highly pathogenic influenza A virus infection [22]. IL-15 activates the effector function of memory phenotype CD8^+ ^cells [23]. In our study, IL-15 is significantly higher in the nvA(H1N1)-ARDS group versus the nvA(H1N1)-mild disease group, but without significant difference in the nvA(H1N1)-ARDS versus bacterial-ARDS groups. Similar to our results, IL-15 was a hallmark of critical illness in the Hong Kong and Spanish nvA(H1N1) cytokine studies [8,9]. IL-15 is significantly higher at admission (P1) and 3 days later (P2) in the nvA(H1N1)-ARDS group for nonsurvivors versus survivors, so it might be pathogenic in lung injury influenza A virus infection. Similarly, To and colleagues found IL-15 significantly higher in critical A(H1N1) patients and very significant in the A(H1N1)-ARDS death group [8].

IFNγ is a cytokine of innate and adaptative immunity. Its major functions are activation of macrophages, differentiation of Th1 from T cells, inhibition of the Th17 pathway and control of intracellular pathogens [24]. Bermejo-Martin and colleagues found high systemic levels of IFNγ in hospitalized patients with nvA(H1N1) [9]. In contrast, in the present study there were no differences between the control and study groups. The IFNγ level over time in the nvA(H1N1) ARDS group was higher at admission than 3 days later, without significant difference between survivors versus nonsurvivors.

TNFα is a cytokine of innate immunity. The principal cellular targets and biologic effects include activation of endothelial cells, neutrophil activation, fever, liver synthesis of acute phase proteins, muscle and fat catabolism, and apoptosis of many cell types. In our study, we found highly increased TNFα levels in the nvA(H1N1)-mild disease, nvA(H1N1)-ARDS and bacterial ARDS groups compared to the control group. TNFα is significantly higher in nvA(H1N1)-ARDS versus nvA(H1N1)-mild disease, with similar results being found by To and colleagues and Bermejo-Martin and colleagues [8,9]. This cytokine is also significantly increased in bacterial-ARDS versus nvA(H1N1)-ARDS.

For the groups of patients with nvA(H1N1), according to the time interval between symptom onset and hospital admission, there were no significant differences found for IL-12 and TNFα levels, but there were significant differences for IL-15 and IFNγ, levels being higher when the time interval was between 6 and 14 days. None of our patients were on oseltamivir medication between symptom onset and admission.

Th17 cells are effective in host defense against certain pathogens and tissue inflammation. Th17 mediators for the development of Th17 cells are IL-6, transforming growth factor beta, IL-8, IL-9, IL-17, IL-1 and IL-23.

IL-6 is a cytokine of innate immunity, its principal targets being the liver cells, the β cells and the naïve T cells [25]. Despite the apparently beneficial role that macrophages play in controlling early viral replication, several reports have demonstrated a more deleterious effect of these cells in influenza A viral infections by excessive inflammation in the lung attributed to IL-6 and TNFα [26]. In our study, IL-6 is increased in nvA(H1N1)-ARDS versus nvA(H1N1)-mild disease. Similarly, IL-6 and IL-15 constituted a hallmark of critical illness in the Hong Kong and Spanish nvA(H1N1) cytokine studies [8,9]. In the nvA(H1N1)-ARDS group, the IL-6 serum level is significantly higher at admission than 3 days later. In the same group, IL-6 is significantly higher in nonsurvivors versus survivors at admission and 3 days later, which seems to further contribute to pulmonary damage and death. We found positive correlations between IL-6, IL-15 and IL-8 levels and a longer than 5 days interval between symptom onset and admission, as well as with C-reactive protein, but a negative correlation with the PaO_2_:FiO_2 _ratio, indicating the severity of the disease.

IL-8 is a chemokine of innate immunity. The chemokine's principal biologic effect is chemotaxis, being a major chemokine for neutrophil activation, and migration into tissues [24]. In our study, IL-8 is highly significant in the nvA(H1N1)-ARDS and ARDS bacterial groups versus the control group, but is not significant in mild disease. In contrast, IL-8 was increased in both critical and noncritical nvA(H1N1) hospitalized patients in the Spanish and Hong Kong studies. In our study, IL-8 is higher in nvA(H1N1)-ARDS versus nvA(H1N1)-mild disease and in bacterial ARDS versus nvA(H1N1)-ARDS. The obese patients with nvA(H1N1) disease had a significant level of IL-8. Plasma IL-8 levels are increased in normoglycemic obese subjects, related to fat mass and the TNFα system [27].

IP-10 is a chemokine of innate immunity, and macrophages and dendritic cells are the principal cell source. We found a higher level of IP-10 in nvA(H1N1)-mild disease, nvA(H1N1)-ARDS and bacterial-ARDS groups versus the control group, and no other differences between groups. In the nvA(H1N1)-ARDS group, the IP-10 level is higher at admission than 3 days after admission because of the survivors' cytokine profile. An increased level of IP-10 was found in the Spanish group as early response to nvA(H1N1) infection in both hospitalized and mild patient disease, as in the present study, while in the Hong Kong group IP-10 was significantly higher in critical patients only. In our study, IP-10 levels in nvA(H1N1)-ARDS nonsurvivors remained higher at admission and 3 days later, being not significantly correlated with the clinical outcome. Emphysema was one of our hystopathological findings and thus it might be speculated that a high level of IP-10 in nonsurvivors could be correlated with emphysema. IP-10 released by lung CD41 and CD81 T cells stimulates alveolar macrophage production of matrix metalloproteinase-12, which digests lung elastin [28,29].

IL-17 is a cytokine of adaptative immunity. Principal cellular targets include endothelial cells with increased chemokine production and macrophages with increased chemokine and cytokine production. This cytokine's principal biologic effect is proinflammatory [24,25]. In the present study IL-17 is significantly higher in the bacterial ARDS group versus the control group and is higher in the bacterial ARDS group versus the nvA(H1N1)-ARDS group. No significant differences between nvA(H1N1)-mild disease versus controls and between nvA(H1N1)-ARDS versus controls were found. In the nvA(H1N1)-ARDS group, IL-17 was higher at admission and lower 3 days later. In the Spanish study the IL-17 level was increased in hospitalized noncritical patients, and in the Hong Kong study no differences between groups were found, similar to the present study.

IL-9, like IL-6, is a Th2 cytokine that induces differentiation of Th17 cells and has anti-inflammatory properties. IL-9 is a cytokine of current interest associated with allergic Th2 responses and is a key modulator of antiviral immunity [30]. In our study IL-9 is significantly higher in the H1N1-ARDS group versus the control group, and is not significantly increased in mild disease - in contrast to the Spanish study, where IL-9 was increased in both critical and noncritical hospitalized patients.

Regarding the behavior of Th17 mediators in nvA(H1N1) groups of patients according to the time interval between symptom onset and admission, there were no differences for IL-9, IL-17 and IP-10 and there were significant differences for IL-6 and IL-8, the levels being higher when the interval was between 6 and 14 days. All our patients with ARDS disease were on corticosteroid treatment, because deficient corticosteroid-mediated downregulation of inflammatory cytokine transcription in ARDS patients is associated with disease progression and mortality. Many studies reported that prolonged corticosteroid treatment was associated with a significant reduction in markers of systemic inflammation [31,32]. In the present study the blood samples for cytokine measurements were taken at admission for the bacterial-ARDS group of patients, and at admission and 3 days later for the nvA(H1N1) group of patients - for this reason, corticosteroid could not significantly affect cytokine levels.

The small number of patients enrolled in the mild disease group is one of our study limitations. Among hospitalized patients with mild flu-like syndrome, only those with risk of severe complications and of secondary outbreaks in the exposed population were sampled for real-time PCR. On the contrary, the laboratory of the National Influenza Centre of Cantacuzino Institute, Bucharest was overwhelmed, being the only centre for influenza PCR diagnosis. Another limitation is the exclusion of children, an important group with nvA(H1N1) virus infection.

## Conclusions

In our critically ill patients with nvA(H1N1) virus infection we found increased levels of some cytokines: IP-10, TNFα, IL-15, IL-12, IL-6, IL-8 and IL-9. The hallmarks for the severity of the disease were IL-6, IL-15, IL-8 and TNFα. We found a positive correlation of IL-6, IL-15 and IL-8 with the admission delay and C-reactive protein and a negative correlation with the PaO_2_:FiO_2 _ratio. The obese patients with nvA(H1N1) disease had a significant level of IL-8. There were significant differences in the level of cytokines when comparing viral ARDS with bacterial ARDS.

## Key messages

• In the influenza-related ARDS group, the levels of IL-6, IL-8, IL-9, IL-12, IL-15, IP-10 and TNFα are significantly increased versus the control group. In the bacterial sepsis-ARDS group, levels of IL-6, IL-8, IL-9, IL-15, IL-17, IP-10 and TNFα are also increased versus the control group. When comparing these two groups, the levels of IL-8, IL-17 and TNFα are significantly higher in bacterial ARDS versus viral ARDS, and IL-12 is increased only in viral ARDS whereas IL-17 is increased only in bacterial ARDS. When comparing the mild nvA(H1N1) and critical ARDS influenza A groups, IL-6, IL-8, IL-15 and TNFα are significantly higher in critical ARDS patients being hallmarks of disease severity.

• The serum levels of IL-15, IL-6, IL-8 and IFNγ according to the interval between symptom onset and admission in hospitalized nvA(H1N1) patients are significantly higher when this interval is longer than 5 days.

• In nonsurvivors versus survivors from the nvA(H1N1)-ARDS group, IL-6 and IL-15 are increased at admission and stay higher 3 days later - which seems to further contribute to pulmonary damage and death.

• There is a positive correlation of IL-6, IL-8 and IL-15 levels with C-reactive protein and with > 5-day interval between symptom onset and hospital admission, and a negative correlation with the PaO_2_:FiO_2 _ratio.

• The obese patients versus nonobese patients with nvA(H1N1) infection have a significant level of IL-8.

## Abbreviations

APACHE: Acute Physiology and Chronic Health Evaluation; ARDS: acute respiratory distress syndrome; ELISA: enzyme-linked immunosorbent assay; ICU: intensive care unit; IFN: interferon; IL: interleukin; IP-10: interferon-inductible protein-10; nvA(H1N1): novel A(H1N1) virus; PaO_2_/FiO_2_: pressure of oxygen in arterial blood/fraction of inspired oxygen; PCR: polymerase chain reaction; SOFA: Sepsis-related Organ Failure Assessment; Th: T-helper type; TNF: tumor necrosis factor.

## Competing interests

The authors declare that they have no competing interests.

## Authors' contributions

NH and AS designed the study, coordinated patient recruitment, supervised laboratory works and wrote the article. DNG and SO performed cytokine profiling and wrote the report. DSD, ESB and MMa collected clinical and laboratory data, and wrote the report. CC assisted in the design of the study and assisted in writing the paper. MMl, RLG and CL supervised clinical aspects, participated in patient recruitment. RH contributed to the statistical analysis. MP provided pulmonary histopathological analysis. DMS assisted in the design of the study, coordinated patient recruitment, analyzed and interpreted the data. All authors read and approved the final manuscript.

## References

[B1] Writing Committee of the WHO Consultation on Clinical Aspects of Pandemic (H1N1) 2009 influenza Clinical aspects of pandemic 2009 influenza A (H1N1) virus infection N Engl J Med 2010 362 1708 1719 10.1056/NEJMra1000449 20445182

[B2] Louie JK Acosta M Winter K Jean C Gavali S Schechter R Vugia D Harriman K Matyas B Glaser CA Samuel MC Rosenberg J Talarico J Hatch D California Pandemic (H1N1) Working Group Factors associated with death or hospitalization due to pandemic 2009 influenza A(H1N1) infection in California JAMA 2009 302 1896 1902 10.1001/jama.2009.1583 19887665

[B3] Jain S Kamimoto L Bramley AM Schmitz AM Benoit SR Louie J Sugerman DE Druckenmiller JK Ritger KA Chugh R Jasuja S Deutscher M Chen S Walker JD Duchin JS Lett S Soliva S Wells EV Swerdlow D Uyeki TM Fiore AE Olsen SJ Fry AM Bridges CB Finelli L 2009 Pandemic Influenza A (H1N1) Virus Hospitalizations Investigation Team Hospitalized patients with 2009 H1N1 influenza in the United States, April-June 2009 N Engl J Med 2009 361 1935 1944 10.1056/NEJMoa0906695 19815859

[B4] Dominguez-Cherit G Lapinsky SE Macias AE Pinto R Espinoza-Perez L de la Torre A Poblano-Morales M Baltazar-Torres JA Bautista E Martinez A Martinez MA Rivero E Valdez R Ruiz-Palacios G Hernandez M Stewart TE Fowler RA Critically ill patients with 2009 influenza A(H1N1) in Mexico JAMA 2009 302 1880 1887 10.1001/jama.2009.1536 19822626

[B5] The ANZIC Influenza Investigators Critical care services and 2009 H1N1 influenza in Australia and New Zealand N Engl J Med 2009 361 1925 1934 10.1056/NEJMoa0908481 19815860

[B6] Kumar A Zarychanski R Pinto R Cook DJ Marshall J Lacroix J Stelfox T Bagshaw S Choong K Lamontagne F Turgeon AF Lapinsky S Ahern SP Smith O Siddiqui F Jouvet P Khwaja K McIntyre L Menon K Hutchinson J Hornstein D Joffe A Lauzier F Singh J Karachi T Wiebe K Olafson K Ramsey C Sharma S Dodek P Canadian Critical Care Trials Group H1N1 Collaborative Critically ill patients with 2009 influenza A(H1N1) infection in Canada JAMA 2009 302 1872 1879 10.1001/jama.2009.1496 19822627

[B7] Pop-Vicas A Rello J Clinical review: primary influenza viral pneumonia Crit Care 2009 13 235 240 10.1186/cc8183 20085663 PMC2811908

[B8] To KK Hung IF Li IW Lee KL Koo CK Yan WW Liu R Ho KY Chu KH Watt CL Luk WK Lai KY Chow FL Mok T Buckley T Chan JF Wong SS Zheng B Chen H Lau CC Tse H Cheng VC Chan KH Yuen KY Pandemic H1N1 Study Group Delayed clearance of viral load and marked cytokine activation in severe cases of pandemic H1N1 2009 influenza virus infection Clin Infect Dis 2010 50 850 859 10.1086/650581 20136415 PMC7107930

[B9] Bermejo-Martin JF de Lejarazu RO Pumarola T Rello J Almansa R Ramirez P Martin-Loeches I Varillas D Gallegos MC Seron C Micheloud D Gomez JM Tenorio-Abreu A Ramos MJ Molina ML Huidobro S Sanchez E Gordon M Fernandez V del Castillo A Marcos MA Villanueva B Lopez CJ Rodriguez-Dominguez M Galan JC Canton R Lietor A Rojo S Eiros JM Hinojosa C Th1 and Th17 hypercytokinemia as early host response signature in severe pandemic influenza Crit Care 2009 13 R201 10.1186/cc8208 20003352 PMC2811892

[B10] Bernard GR Artigas A Brigham KL Carlet J Falke K Hudson L Lamy M Legall JR Morris A Spragg R The American-European Consensus Conference on ARDS. Definition, mechanisms, relevant outcome, and clinical trial coordination Am J Respir Crit Care Med 1994 149 818 824 7509706 10.1164/ajrccm.149.3.7509706

[B11] Offner PJ Moore EE Lung injury severity scoring in the era of lung protective mechanical ventilation: the PaO_2_/FiO_2 _ratio J Trauma 2003 55 285 289 10.1097/01.TA.0000078695.35172.79 12913639

[B12] Gill JR Sheng ZM Ely SF Guinee DG Beasley MB Suh J Deshpande C Mollura DJ Morens DM Bray M Travis WD Taubenberger JK Pulmonary pathologic findings of fatal 2009 pandemic influenza A/H1N1 viral infections Arch Pathol Lab Med 2010 134 235 243 20121613 10.5858/134.2.235 PMC2819217

[B13] Mauad T Hajjar LA Callegari GD da Silva LF Schout D Galas FR Alves VA Malheiros DM Auler JO Jr Ferreira AF Borsato MR Bezerra SM Gutierrez PS Caldini ET Pasqualucci CA Dolhnikoff M Saldiva PH Lung pathology in fatal novel human influenza A(H1N1) infection Am J Respir Crit Care Med 2010 181 72 79 10.1164/rccm.200909-1420OC 19875682

[B14] Romagnani S T-cell subsets (Th1 versus Th2) Ann Allergy Asthma Immunol 2000 85 9 18 10.1016/S1081-1206(10)62426-X 10923599

[B15] Hamza T Barnett JB Li B Interleukin 12 a key immunoregulatory cytokine in infection applications Int J Mol Sci 2010 11 789 806 10.3390/ijms11030789 20479986 PMC2869233

[B16] Palmer EM Farrokh-Siar L Maguire van Seventer J van Seventer GA IL-12 decreases activation-induced cell death in human naïve Th cells costimulated by intercellular adhesion molecule-1.I. IL-12 alters caspase processing and inhibits enzyme function J Immunol 2001 167 749 758 11441079 10.4049/jimmunol.167.2.749

[B17] Pirtskhalaishvili G Shurin GV Esche C Cai Q Salup RR Bykovskaia SN Lotze MT Shurin MR Cytokine-mediated protection of human dendritic cells from prostate cancer-induced apoptosis is regulated by the Bcl-2 family of proteins Br J Cancer 2000 83 506 513 10.1054/bjoc.2000.1289 10945499 PMC2374651

[B18] Grohmann U Belladonna ML Vacca C Bianchi R Fallarino F Orabona C Fioretti MC Puccetti P Positive regulatory role of IL-12 in macrophages and modulation by IFN-gamma J Immunol 2001 167 221 227 11418652 10.4049/jimmunol.167.1.221

[B19] Brydon EW Smith H Sweet C Influenza A virus-induced apoptosis in bronchiolar epithelial (NCI-H292) cells limits pro-inflammatory cytokine release J Gen Virol 2003 84 2389 2400 10.1099/vir.0.18913-0 12917460

[B20] Berard M Brandt K Bulfone Paus S Tough D IL-15 promotes the survival of naïve and memory phenotype CD8^+ ^T cells J Immunol 2003 170 5018 5026 12734346 10.4049/jimmunol.170.10.5018

[B21] Yajima T Yoshihara K Nakazato K Kumabe S Koyasu S Sad S Shen H Kuwano H Yoshikai Y IL-15 regulates CD8^+ ^T cell contraction during primary infection J Immunol 2006 176 507 515 16365444 10.4049/jimmunol.176.1.507

[B22] Nakamura R Maeda N Shibata K Yamada H Kase T Yoshikai Y Interleukin-15 is critical in the pathogenesis of influenza a virus-induced acute lung injury J Virol 2010 84 5574 5582 10.1128/JVI.02030-09 20335267 PMC2876592

[B23] Liu K Catalfamo M Li Y Henkart PA Weng NP IL-15 mimics T cell receptor crosslinking in the induction of cellular proliferation, gene expression, and cytotoxicity in CD8^+ ^memory T cells Proc Natl Acad Sci USA 2002 99 6192 6197 10.1073/pnas.092675799 11972069 PMC122925

[B24] Miossec P Korn T Kuchroo VK Interleukin-17 and Type 17 helper T cells N Engl J Med 2009 361 888 898 10.1056/NEJMra0707449 19710487

[B25] Korn T Oukka M Kuchroo V Bettelli E Th17 cells: effector T cells with inflammatory properties Semin Immunol 2007 19 362 371 10.1016/j.smim.2007.10.007 18035554 PMC2839934

[B26] McGill J Heusel JW Legge KL Innate immune control and regulation of influenza virus infections J Leukoc Biol 2009 86 803 812 10.1189/jlb.0509368 19643736 PMC2752015

[B27] Straczkowski M Straczkowska SD Stepien A Kowalska I Szelachowska M Kinalska I Plasma interleukin-8 concentrations are increased in obese subjects and related to fat mass and tumor necrosis factor-α system J Clin Endocrinol Metab 2002 87 4602 4606 10.1210/jc.2002-020135 12364441

[B28] Barnes PJ Shapiro SD Pauwels RA Chronic obstructive pulmonary disease: molecular and cellular mechanisms Eur Respir J 2003 22 672 688 10.1183/09031936.03.00040703 14582923

[B29] Grumelli S Corry DB Song LZ Song L Green L Huh J Hacken J Espada R Bag R Lewis DE Kheradmand F An immune basis for lung parenchymal destruction in chronic obstructive pulmonary disease and emphysema PLoS Med 2004 1 e8 10.1371/journal.pmed.0010008 15526056 PMC523885

[B30] Dodd JS Lum E Goulding J Muir R Van Snick J Openshaw PJM *IL*-9 regulates pathology during primary and memory responses to respiratory syncytial virus infection J Immunol 2009 183 7006 7013 10.4049/jimmunol.0900085 19915054

[B31] Meduri GU Tolley EA Chrousos GP Stentz F Prolonged methylprednisolone treatment suppresses systemic inflammation in patients with unresolving acute respiratory distress syndrome. Evidence for inadequate endogenous glucocorticoid secretion and inflammation-induced immune cell resistance to glucocorticoids Am J Respir Crit Care Med 2002 165 983 991 11934726 10.1164/ajrccm.165.7.2106014

[B32] Annane D Bellissant E Bollaert PE Briegel J Confalonieri M De Gaudio R Keh D Kupfer Y Oppert M Meduri GU Corticosteroids in the treatment of severe sepsis and septic shock in adults: a systematic review JAMA 2009 301 2362 2375 10.1001/jama.2009.815 19509383

